# *Lactobacillus plantarum* Ameliorates High-Carbohydrate Diet-Induced Hepatic Lipid Accumulation and Oxidative Stress by Upregulating Uridine Synthesis

**DOI:** 10.3390/antiox11071238

**Published:** 2022-06-24

**Authors:** Rong Xu, Tong Wang, Fei-Fei Ding, Nan-Nan Zhou, Fang Qiao, Li-Qiao Chen, Zhen-Yu Du, Mei-Ling Zhang

**Affiliations:** School of Life Sciences, East China Normal University, Shanghai 200241, China; 52181300029@stu.ecnu.edu.cn (R.X.); 52211300035@stu.ecnu.edu.cn (T.W.); 52201300060@stu.ecnu.edu.cn (F.-F.D.); 51201300089@stu.ecnu.edu.cn (N.-N.Z.); fqiao@bio.ecnu.edu.cn (F.Q.); lqchen@bio.ecnu.edu.cn (L.-Q.C.)

**Keywords:** high-carbohydrate diet, *Lactobacillus plantarum*, oxidative stress, acetate, uridine

## Abstract

The overconsumption of carbohydrates induces oxidative stress and lipid accumulation in the liver, which can be alleviated by modulation of intestinal microbiota; however, the underlying mechanism remains unclear. Here, we demonstrated that a strain affiliated with *Lactobacillus plantarum* (designed as MR1) efficiently attenuated lipid deposition, oxidative stress, as well as inflammatory response, which are caused by high-carbohydrate diet (HC) in fish with poor utilization ability of carbohydrates. Serum untargeted metabolome analysis indicated that pyrimidine metabolism was the significantly changed pathway among the groups. In addition, the content of serum uridine was significantly decreased in the HC group compared with the control group, while it increased by supplementation with *L. plantarum* MR1. Further analysis showed that addition of *L. plantarum* MR1 reshaped the composition of gut microbiota and increased the content of intestinal acetate. In vitro experiment showed that sodium acetate could induce the synthesis of uridine in hepatocytes. Furthermore, we proved that uridine could directly ameliorate oxidative stress and decrease liver lipid accumulation in the hepatocytes. In conclusion, this study indicated that probiotic *L. plantarum* MR1 ameliorated high-carbohydrate diet-induced hepatic lipid accumulation and oxidative stress by increasing the circulating uridine, suggesting that intestinal microbiota can regulate the metabolism of nucleotides to maintain host physiological homeostasis.

## 1. Introduction

Over intake of high-carbohydrate diet promotes the oxidative stress and subsequently induces the inflammatory response in animals [1,2]. Moreover, it will increase the de novo lipogenesis. Oxidative stress and lipid accumulation contribute to metabolic syndrome [3,4], including fatty liver [5], high glucose level, and impaired insulin function in animals [6]. Postprandial oxidative stress is regarded as a secondary response to postprandial high glucose and triglyceride levels [7], and oxidative stress may be the mechanistic link between lipid metabolism and related complications [8]. Therefore, the study to attenuate lipid accumulation and oxidative stress induced by high-carbohydrate diet has attracted increased attention.

Carbohydrates are important energy sources for fish; however, fish are natural glucose intolerant with poor utilization ability of carbohydrates [9,10]. High dietary carbohydrates cause negative effects on the growth performance and liver health, and easily trigger oxidative stress in fish. For example, it has been reported that high-carbohydrate diet induces de novo fatty acid synthesis, insulin resistance, and fatty liver disease in blunt snout bream (*Megalobrama amblycephala*) [11]. High-carbohydrate diet affects the growth performance, hepatic glucose metabolism, and antioxidant capacity of largemouth bass (*Micropterus salmoides*) [12]. Fish has become an important model organism to study the host physiological mechanisms [13,14], particularly for glucose metabolism [15].

Gut microbiota has been considered as an important organ with properties involving host metabolism and physiological functions [16]. It has been found that gut microbiota dysbiosis is strongly correlated with hepatic lipid metabolism disorder or oxidative stress [17,18], while gut microbiota homeostasis restoration can prevent diet-induced fatty liver diseases, glucose metabolism disorder or other metabolic syndromes [19]. Therefore, intestinal microbiota indeed acts as a mediator in the lipid metabolism dysregulation and oxidative stress development [20,21]. It has been found that *Bifidobacterium lactis HN019* has a beneficial effect on the regulation of inflammatory and oxidative biomarkers in subjects with the metabolic syndrome [22]. In addition, *Lactobacillus plantarum* can exert a beneficial effect in high-fat/high-fructose diet-induced fatty liver disease in rats [23], and attenuate oxidative stress by regulating the composition of gut microbiota in D-galactose-exposed mice [24,25]. However, the mechanisms by which microbes regulate lipid accumulation or activate host antioxidant responses still require further investigation. A recent study found that *Lactobacillus rhamnosus* GG activated hepatic nuclear factor erythroid-2-related factor 2 (Nrf2) signal by producing 5-methoxyindoleacetic acid in Drosophila melanogaster and mice, suggesting that bacteria-derived molecules can protect against liver oxidative injury [26].

The short-chain fatty acids (SCFAs) produced by bacterial fermentation of dietary fiber in the gut play critical roles in glucose and lipid metabolism or oxidative stress modulation [27]. For example, butyrate has been reported to exert an antioxidant effect by activating Nrf2 and H3K9/14 acetylation via G-protein coupled receptor (GPR) 109A in bovine mammary epithelial cells [28]. Propionate induces intestinal oxidative stress resulting from Sod2 propionylation in zebrafish fed with high-fat diet (HFD) [29]. Acetate, the most abundant SCFAs in the gut, can regulate lipid metabolism, improve glucose intolerance and insulin resistance, and inhibit the inflammatory response by activating GPRs [27,30]. Accordingly, except for acting as signal molecules, SCFAs can be used as a substrate to synthesize some precursor substances, and then participate in lipid metabolism. However, the related mechanisms need to be further elucidated [31,32].

Nile tilapia (*Oreochromis niloticus*) is the second most farming fish species worldwide, which is also an important model for the physiological study of fish with available genomic information [33,34]. In this study, a strain affiliated with *L. plantarum* (MR1) was isolated from the intestine of healthy Nile tilapia. Three diets, including a normal control diet (NC), a high-carbohydrate diet (HC), and a high-carbohydrate diet supplemented with *L. plantarum* MR1 (HCL), were used to feed the fish for 10 weeks. Then, the effect of *L. plantarum* MR1 on the growth performance, liver lipid metabolism, inflammatory response, and oxidative stress in the HC-fed Nile tilapia was analyzed. The possible mechanism was identified by serum untargeted metabolome, gut microbiome analysis, and in vitro experiment. The present study revealed the method by which intestinal microbiota regulates the metabolism of nucleotides to maintain host physiological homeostasis.

## 2. Materials and Methods

### 2.1. Animal Ethics

Experiments were conducted under the Guidance of the Care and Use of Laboratory Animals in China. This study was approved by the Committee on the Ethics of Animal Experiments of East China Normal University (F20190101).

### 2.2. Animal Experiments

A strain affiliated with *L. plantarum*, designed as MR1, was purified from the gut of healthy Nile tilapia (additional details in the Supplementary Methods).

Nile tilapia juveniles were obtained from Yueqiang aquafarm (Guangzhou, China). All fish were acclimated to 28 °C and fed with a commercial diet twice per day for 2 weeks. Following the acclimation, 270 similar-sized fish at 1.66 (± 0.05) g were randomly distributed into three groups (three replicates for each group, thirty fish per replicate) and fed with a normal control diet (NC), a high-carbohydrate diet (HC) or a high-carbohydrate diet supplemented with *L. plantarum* MR1 (HCL) for 10 weeks. All fish were fed twice per day at 5% of body weight. The formulations of the diets were listed in Table S1. The total weight of fish in each tank was recorded every 2 weeks. For additional details on sampling collection, see Supplementary Methods.

### 2.3. Body Composition Assessment

Nine fish from each group were collected for the body composition assay. The whole fish were dried in an electric oven at 105 °C to a constant weight. The dried fish were ground to mince for determination of total protein analysis using a semi-automatic Kjeldahl System (Kjeltec 8200, FOSS, Hillerod, Denmark) and for total lipid content detection by a classic methanol–chloroform method. The ash was determined by incineration in a muffle furnace at 550 °C to a constant weight.

### 2.4. Biochemical Analysis

Serum samples were directly used for the biochemical detection. Liver samples from fish were homogenized with 1 × PBS to obtain a 10% (*w/v*) liver homogenate, centrifuged at 4 °C (3500× *g* for 10 min) for further experiments. Aspartate aminotransferase (AST, C010-2-1), alanine aminotransferase (ALT, C009-2-1), triglyceride (TG, A110-1-1), non-esterified free fatty acids (NEFA, A042-2-1), superoxide dismutase (SOD, A001-3-2), malondialdehyde (MDA, A003-1-2), and reduced glutathione (GSH, A006-2-1) were detected using biochemical assay kits, in accordance with the manufacturer’s instructions (Nanjing Jiancheng Bioengineering Institute, Nanjing, China).

### 2.5. Histological Analysis

Liver tissues were fixed in 4% paraformaldehyde followed by gradient ethanol dehydration and xylene transparency procedure. In addition, the tissues were embedded in paraffin, which were then sliced into 5 μm for hematoxylin and eosin (H&E) staining. For oil red O staining, liver tissues were immediately frozen at −80 °C using the OCT embedding agent (G6059-110ML, Servicebio, Wuhan, China). Approximately 5–10 μm frozen slices were stained with oil red O (ORO) for 10 min in the dark, and then immersed in 60% isopropanol for a few seconds. The frozen slices were counterstained with hematoxylin to visualize lipid droplets. The histological features were observed and captured under a Nikon Eclipse Ti-SR inverted microscope (Nikon, Japan). Quantification and statistical analysis were conducted using Image J v.1.8.0 (National Institutes of Health, Bethesda, MD, USA).

### 2.6. 16S rRNA Amplicon Sequencing

Intestinal bacterial genomic DNA was purified using an E.Z.N.A.^®^ Soil DNA Kit (D5625, Omega, Norcross, GA, USA), in accordance with the manufacturer’s instructions. The V3-V4 region of bacterial 16S rRNA gene was amplified by PCR and sequenced on an Illumina MiSeq PE300 platform/NovaSeq PE250 platform (Illumina, San Diego, CA, USA). For additional details, see Supplementary Methods.

### 2.7. Measurement of SCFAs

Quantification of bacterial and intestinal SCFAs (including acetate, propionate, and butyrate) was conducted using the previously described methods [35]. For additional details, see Supplementary Methods.

### 2.8. Mass Spectrometry

Serum untargeted metabolome was analyzed by liquid chromatography–mass spectrometry (LC–MS). Chromatographic separation of the metabolites was performed on a Thermo UHPLC system equipped with an ACQUITY UPLC HSS T3 (100 × 2.1 mm i.d., 1.8 µm, Waltham, MA, USA) column. For additional details on the detection of all the samples, see Supplementary Methods.

### 2.9. Cell Culture

The primary hepatocytes were generated from healthy Nile tilapia, as previously described [36]. Briefly, the liver tissues were washed three times using 1 × PBS and digested with DMEM containing 0.1% collagenase IV (17104019, Gibco, Suwanee, GA, USA), then filtered through a 70 µm-nylon mesh. The hepatocytes were harvested by centrifuging at 850× *g* for 10 min. Red blood cells were removed using a red cell lysis buffer (RT122-02, Tiangen, Beijing, China), and the remaining cells were incubated in DMEM with 10% FBS and 1% penicillin–streptomycin at 28 °C. To explore whether acetate acted as the substrate for uridine synthesis, 20 mM sodium acetate (NaAc) (S5636, Sigma-Aldrich, St. Louis, MO, USA) was added to the hepatocytes for 24 h to detect the concentration of uridine. For additional details on the effects of uridine in oleic acid (OA) and hydrogen peroxide (H_2_O_2_)-treated hepatocytes, see Supplementary Methods.

### 2.10. Uridine and Acetyl-CoA Detection by HPLC

Uridine and acetyl-CoA were detected by high performance liquid chromatography (HPLC) (LC-20AT, Shimadzu, Takamatsu, Kagawa, Japan) with a tandem double plunger. The separation was achieved on the Shim-pack GIST 5 μm C18 column (4.6 × 250 μm, 5 μm particle size, Shimadazu^®^, Takamatsu, Kagawa, Japan). For additional details on the contents of serum uridine and liver acetyl-CoA analysis, see Supplementary Methods.

### 2.11. Real-Time Quantitative PCR (RT-qPCR)

Real-time quantitative PCR (RT-qPCR) was performed as previously described [35]. Briefly, total RNA was extracted, and cDNAs were transcribed using RNAs as templates. The cDNAs were amplified by PCR with ChamQ Universal SYBR qPCR Master Mix (Q711, Vazyme, Nanjing, China) using the primers listed in Table S2. Real-time quantitative PCR (RT-qPCR) analyses were performed using CFX Connect (Bio-rad, Richmond, CA, USA). The *β-actin* and *ef-1α* were used as house-keeping genes, and the relative gene expression values were quantified using the 2^−ΔΔCT^ method.

### 2.12. Western Blot Analysis

Tissues or cells were lysed, and total protein contents were extracted and quantified. Proteins were subjected to SDS-PAGE and transferred to NC membrane, then blocked with 5% BSA for 1 h at room temperature. The membrane was incubated with primary antibodies overnight at 4 °C. Following the secondary antibody incubation, the protein bands were visualized in the Odyssey CLx Imager (Li-Cor Biotechnology, Lincoln, NE, USA) and quantified using Image J v.1.8.0. GAPDH was served as a reference protein. The antibodies used in this study were listed in Table S3.

### 2.13. Statistical Analysis

Statistical analysis of all data was performed using GraphPad Prism 7.0. The results of biological assays are presented as mean ± standard error of the mean (SEM). Datasets were assessed using one-way analysis of variance (ANOVA) with Tukey’s adjustment. Differences between two groups were analyzed by Student’s *t*-test. In the Figures: *, *p* < 0.05; **, *p <* 0.01; ***, *p <* 0.001.

## 3. Results

### 3.1. L. plantarum MR1 Promotes Growth Performance and Decreases Lipid Accumulation of Nile Tilapia

At the end of the feeding experiment, the growth performance showed that final body weight (FBW) and weight gain rate (WGR) (Table 1) were significantly increased in the HC group compared with the NC group, and the WGR was further increased by addition of *L. plantarum* MR1 (Table 1). The body composition indexes showed that the visceral somatic index (VSI) and hepatosomatic index (HSI) were markedly increased in the HC group compared with the NC group; however, they were decreased in the HCL group when compared with the HC group (Table 1). Although there was no significant difference, the mesenteric fat index (MFI) was higher in the HC group than the NC group, and it had a declining tendency in the HCL group, compared with the HC group (Table 1). Compared with the HC-fed fish, the carcass index (CI) was significantly increased in *L. plantarum* MR1-treated fish (Table 1). The total lipid contents of the whole fish were clearly increased in the HC-fed fish, but significantly decreased by *L. plantarum* MR1 administration without influencing the total protein contents (Table 1). These results suggested that *L. plantarum* MR1 could promote the growth performance and decrease the total lipid contents in Nile tilapia.

### 3.2. L. plantarum MR1 Reduces HC-Induced Liver Lipid Deposition of Nile Tilapia

**Figure 1:**
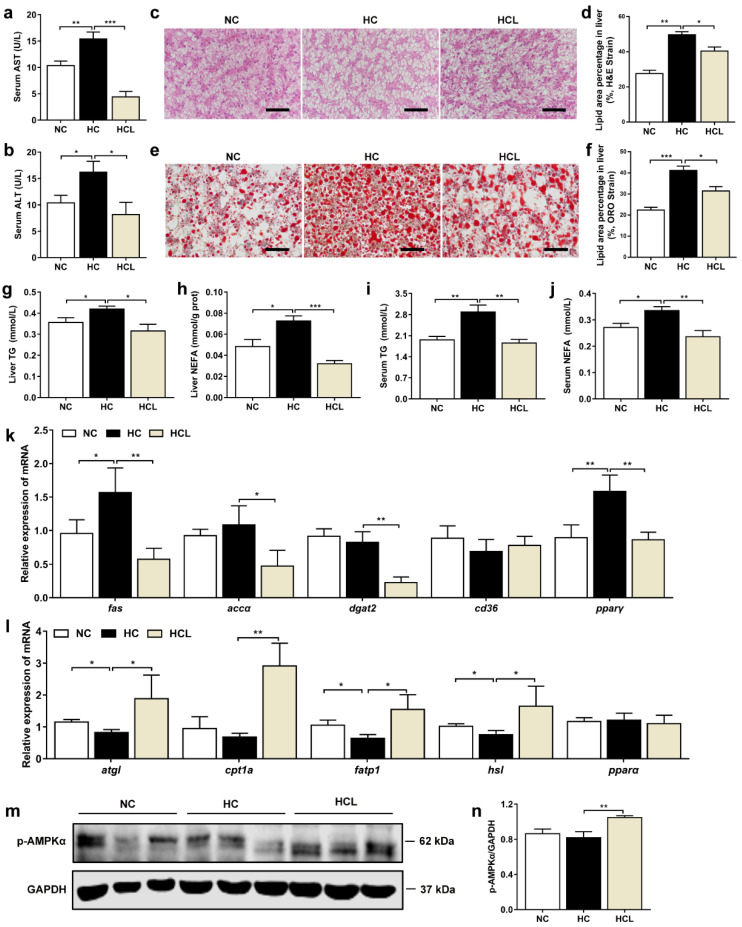
*L. plantarum* MR1 improved liver health in Nile tilapia fed with high-carbohydrate diet. (**a**,**b**) Serum contents of aspartate aminotransferase (AST) (**a**) and alanine aminotransferase (ALT) (**b**) (*n* = 6). (**c**–**f**) Examination of the liver condition by hematoxylin and eosin (H&E) staining (**c**,**d**) and oil red O (ORO) staining (**e**,**f**). Scale bar, 50 µm (*n* = 3 slices). (**g**–**j**) Detection of the liver and serum triglyceride (TG) and non-esterified free fatty acid (NEFA) levels (*n* = 6). (**k**) Relative expression of lipid synthesis genes in the liver (*n* = 6). (**l**) Relative expression of lipid catabolism genes in the liver (*n* = 6). (**m**) Protein level of phosphorylated-AMP-activated protein kinase alpha (p-AMPKα) energy homeostasis regulator in the liver (*n* = 3). (**n**) The protein level of p-AMPKα was quantified and normalized to the loading control (GAPDH) (*n* = 3). Statistics were analyzed by one-way ANOVA with Tukey’s adjustment and presented as mean ± SEM (*, *p <* 0.05; **, *p <* 0.01; ***, *p <* 0.001).

Compared with the NC group, the levels of serum aspartate aminotransferase (AST) and alanine aminotransferase (ALT) (markers of liver injury) were considerably higher in the HC group; however, they were clearly reduced by supplementation with *L. plantarum* MR1 (Figure 1a,b). The hematoxylin and eosin (H&E) staining displayed that the hepatic vacuolation was significantly increased in the HC group when compared with the NC group; however, it was decreased by addition of *L. plantarum* MR1 (Figure 1c,d). Moreover, liver oil red O (ORO) staining showed that HC-fed fish had the highest lipid accumulation, which was significantly prevented by addition of *L. plantarum* MR1 (Figure 1e,f). Further analysis also indicated that the contents of triglyceride (TG) and non-esterified free fatty acids (NEFA) in liver and serum were significantly decreased in the fish supplemented with *L. plantarum* MR1 (Figure 1g–j), suggesting that *L. plantarum* MR1 could reduce the HC-induced liver lipid deposition in Nile tilapia.

To investigate the mechanism by which *L. plantarum* MR1 reduces the liver lipid accumulation, the relative expression of genes related to lipid metabolism were detected. The expression levels of genes related to lipid synthesis, such as *fas* and *pparγ*, were significantly higher in the HC group than those in the NC group, and the expression levels of *fas*, *accα*, *dgat2,* and *pparγ,* were considerably lower in the HCL group relative to the HC group (Figure 1k). The genes associated with lipid catabolism, such as *atgl*, *fatp1,* and *hsl*, were downregulated in the HC group compared with the NC group; however, addition of *L. plantarum* MR1 significantly increased the transcript levels of *atgl*, *fatp1*, *cpt1a,* and *hsl* (Figure 1l). To define whether the energy expenditure was increased, the protein expression of AMP-activated protein kinase alpha (AMPKα), a key protein in energy signaling pathway, was analyzed, and a significant increase in phosphorylated-AMPKα (p-AMPKα) was observed in *L. plantarum* MR1-treated group (Figure 1m,n), suggesting that addition of *L. plantarum* MR1 reduced lipid deposition by activating the AMPKα signaling pathway in Nile tilapia.

Lipid accumulation in the liver normally causes inflammation. The inflammatory cytokines were significantly higher in the HC group than the NC group; however, they were notably lowered in *L. plantarum* MR1-treated group (Figure S1a). In addition, the inflammatory marker proteins, phosphorylated-nuclear factor-kappa B (p-NF-κB), and cleaved interleukin-1 beta (IL-1β), were clearly decreased in the HCL group (Figure S1b,c), indicating that *L. plantarum* MR1 could suppress the HC-induced inflammatory response.

### 3.3. L. plantarum MR1 Attenuates the HC-Induced Oxidative Stress of Nile Tilapia

**Figure 2:**
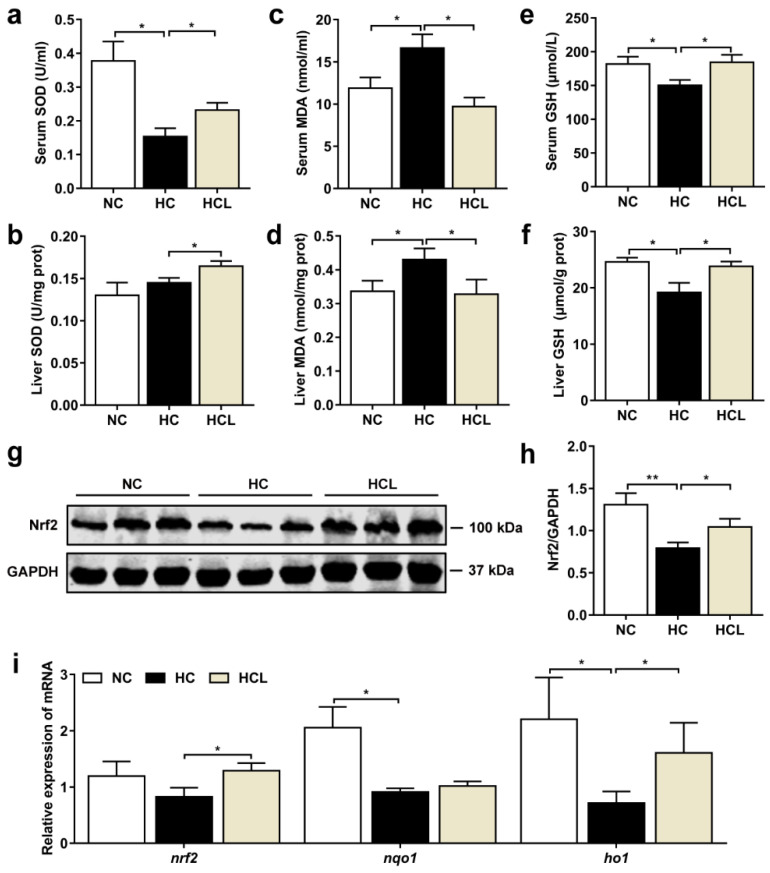
*L. plantarum* MR1 relieved the HC-induced oxidative stress in Nile tilapia. (**a**,**b**) The activities of superoxide dismutase (SOD) in the serum (**a**) and liver (**b**) (*n* = 6). (**c**,**d**) The contents of malondialdehyde (MDA) in the serum (**c**) and liver (**d**) (*n* = 6). (**e**,**f**) The contents of reduced glutathione (GSH) in the serum (**e**) and liver (**f**) (*n* = 6). (**g**,**h**) The protein expression and quantification of Nrf2 in the liver (*n* = 6). (**i**) The relative mRNA expression of genes for Nrf2 signaling pathway in the liver (*n* = 6). Statistics were analyzed by one-way ANOVA with Tukey’s adjustment and presented as mean ± SEM (*, *p <* 0.05; **, *p <* 0.01).

As lipid accumulation may trigger the oxidative stress in the liver, the antioxidant effect of *L. plantarum* MR1 was evaluated. The activity of superoxide dismutase (SOD) was elevated by addition of *L. plantarum* MR1 compared with the HC group (Figure 2a,b). The lipid peroxidation product malondialdehyde (MDA), an oxidative damage marker, was significantly increased in the HC-treated fish compared with the NC-treated fish; however, it was notably reduced by *L. plantarum* MR1 treatment (Figure 2c,d). The contents of reduced glutathione (GSH) were significantly decreased in serum or liver in the HC-fed fish compared with the NC-fed fish; however, they were markedly increased in *L. plantarum* MR1-treated fish (Figure 2e,f). As a master regulator of antioxidative responses, the protein expression of Nrf2 was significantly decreased in the HC group, but increased in the HCL group (Figure 2g,h). The expression levels of genes in Nrf2 signaling pathway, including *nqo1* and *ho1*, were downregulated in the HC group compared with the NC group; however, the expression levels of *nrf2* and *ho1* were upregulated in *L. plantarum* MR1-treated group (Figure 2i). Considered together, these results demonstrated that addition of *L. plantarum* MR1 could attenuate the HC-induced oxidative stress by activating the Nrf2 signaling pathway in Nile tilapia.

### 3.4. L. plantarum MR1 Regulates the Nucleotide Metabolism of Nile Tilapia

**Figure 3:**
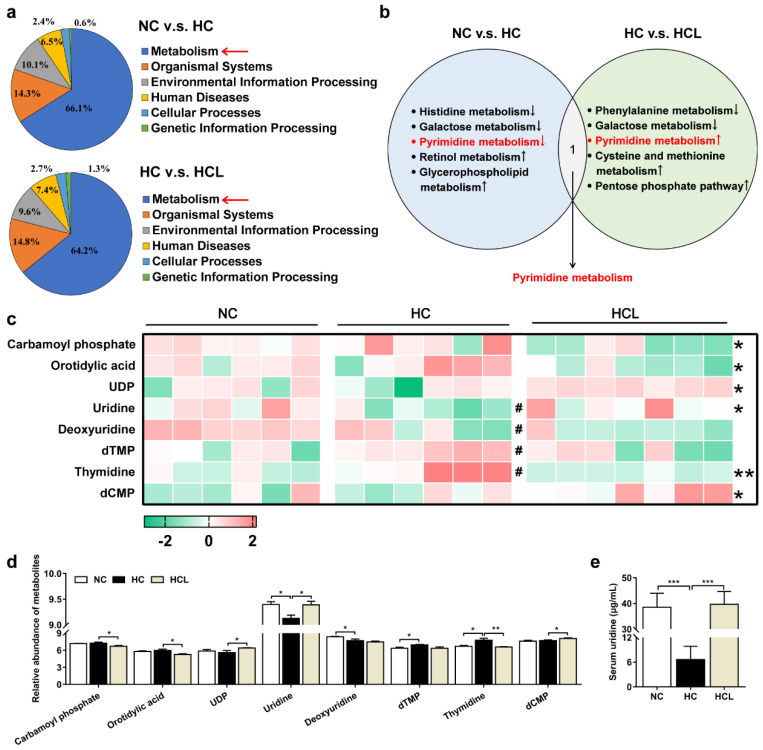
*L. plantarum* MR1 modulated the metabolic patterns in Nile tilapia. (**a**) Identification of differentially biological processes percentage in the HC-treated fish compared with the NC-fed fish or in *L. plantarum* MR1-treated fish compared with the HC-treated fish by liquid chromatography–mass spectrometry (LC–MS) analysis. (**b**) Annotation of the identical significant altered metabolism pathway in the three groups. (**c**) Heat map of selected differentially changed metabolites involved in pyrimidine metabolism (#, NC v.s. HC; *, HC v.s. HCL, #, *p <* 0.05, *, *p <* 0.05, **, *p <* 0.01). (**d**) Histogram statistics of differential metabolites in the heat map. (**e**) The contents of serum uridine detection by high performance liquid chromatography (HPLC); *n* = 6 in NC and HC groups and *n* = 7 in HCL group. Statistics were analyzed by one-way ANOVA with Tukey’s adjustment and presented as mean ± SEM (*, *p <* 0.05; **, *p <* 0.01; ***, *p <* 0.001).

The serum untargeted metabolome analysis was performed to identify the composition of metabolites among the treatments. The results showed that the metabolism process was obviously changed among the groups (NC v.s. HC, 66.1%; HC v.s. HCL, 64.2%) (Figure 3a). Further analysis showed that the change in pyrimidine metabolism was shared by the comparison between NC v.s. HC and HC v.s. HCL (Figure 3b). The heatmap displayed that the metabolite uridine, a pyrimidine nucleotide, was markedly decreased in the HC group, but clearly increased in the HCL group (Figure 3c,d). Moreover, the uridine concentration in the serum was confirmed by high performance liquid chromatography (HPLC), and the result was consistent with the metabolome analysis (Figure 3e). Considered together, these data demonstrated that supplementation of *L. plantarum* MR1 could increase the contents of uridine, which were decreased in the serum of HC-fed Nile tilapia.

### 3.5. Uridine Alleviates the OA-Induced Lipid Accumulation in the Primary Hepatocytes of Nile Tilapia

**Figure 4:**
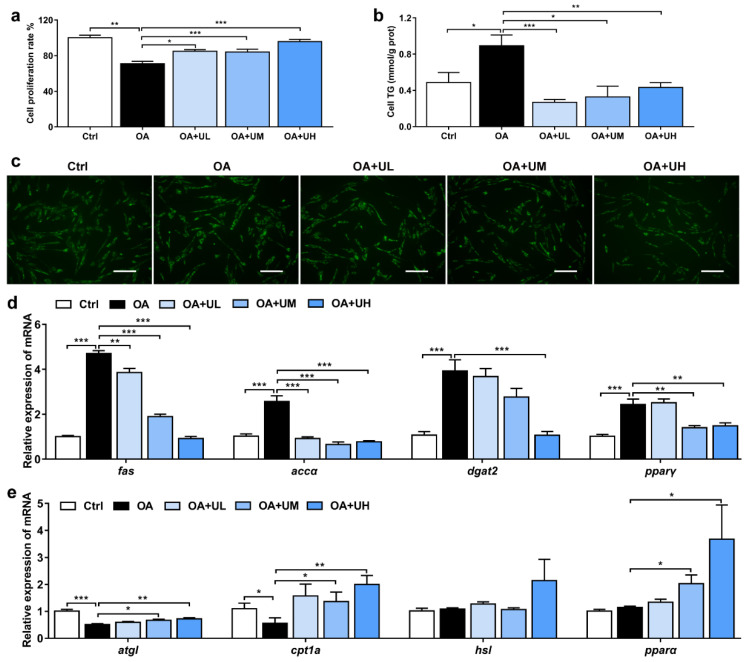
Uridine reduced the OA-induced accumulation of lipid droplets in the primary hepatocytes of Nile tilapia. (**a**) The proliferation of cells that are analyzed by CCK-8 in oleic acid (OA)-induced cell model (*n* = 10). (**b**) The contents of TG in the hepatocytes (*n* = 6). (**c**) Lipid droplets staining with BODIPY 493/503 in the hepatocytes. Scale bar, 100 µm (*n* = 6). (**d**,**e**) Gene expression of lipid synthesis (**d**) and catabolism (**e**) in the hepatocytes (*n* = 6). Statistics were analyzed by one-way ANOVA with Tukey’s adjustment and presented as mean ± SEM (*, *p <* 0.05; **, *p <* 0.01; ***, *p <* 0.001).

To explore the effect of uridine in reducing liver lipid deposition, 250 μM of oleic acid (OA) was added to the primary hepatocytes of Nile tilapia to construct the lipid accumulation model. Uridine at the concentration of 25 μM (OA + UL), 125 μM (OA + UM) or 250 μM (OA + UH) was added to the OA-treated cells. The cell proliferation rate was significantly decreased in the OA group when compared with the Ctrl group; however, it was notably increased by addition of uridine, indicating that no cytotoxicity was caused by uridine (Figure 4a). The contents of TG in the cells were markedly increased in the OA-treated group compared with the Ctrl group; however, they were dramatically reduced by addition of uridine (Figure 4b). Compared with the Ctrl group, the lipid droplets were significantly increased in the OA group, but were clearly diminished by addition of uridine in the hepatocytes, in accordance with BODIPY 493/503 staining (Figure 4c). The relative expression of genes related to lipid synthesis, such as *fas*, *accα*, *dgat2,* and *pparγ*, were significantly upregulated in the OA-treated group relative to the Ctrl group, but were downregulated by supplementation with uridine (Figure 4d). The relative expression of genes associated with lipid catabolism, such as *atgl* and *cpt1a*, was lower in the OA-treated group than the Ctrl group, while the expression of *atgl*, *cpt1a,* and *pparα,* was higher in the uridine administration group (Figure 4e). Considered together, our data suggested that uridine could ameliorate the OA-induced lipid droplets accumulation by increasing lipid catabolism and decreasing lipid synthesis.

Based on our previous assumption, we further evaluated the influence of uridine on inflammatory response. Consistently, the expression of p-NF-κB and cleaved IL-1β was remarkably increased in the OA-treated group compared with the Ctrl group, but notably suppressed by the uridine treatment (Figure S2).

### 3.6. Uridine Ameliorates Oxidative Stress in the Primary Hepatocytes of Nile Tilapia

**Figure 5:**
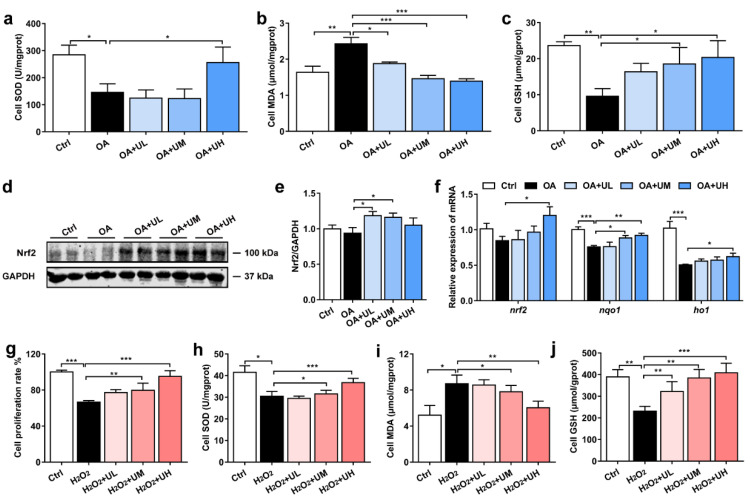
Uridine inhibited oxidative stress in the primary hepatocytes of Nile tilapia. (**a**) The activity of SOD in the primary hepatocytes (*n* = 6). (**b**) The contents of MDA in the primary hepatocytes (*n* = 6). (**c**) The contents of GSH in the primary hepatocytes (*n* = 6). (**d**) The protein expression of Nrf2 in the hepatocytes (*n* = 4). (**e**) Quantitation of Nrf2 levels normalized to GAPDH levels from blots shown in (**d**) (*n* = 4). (**f**) The gene expression of Nrf2 signaling pathway in the hepatocytes (*n* = 6). (**g**) The cells proliferation analyzed by CCK-8 in hydrogen peroxide (H_2_O_2_)-induced cell model (*n* = 10). (**h**) The activity of SOD in the primary hepatocytes (*n* = 6). (**i**) The contents of MDA in the primary hepatocytes (*n* = 6). (**j**) The contents of GSH in the primary hepatocytes (*n* = 6). Statistics were analyzed by one-way ANOVA with Tukey’s adjustment and presented as mean ± SEM (*, *p <* 0.05; **, *p <* 0.01; ***, *p <* 0.001).

We further investigated whether uridine could attenuate OA-induced oxidative injury in the hepatocytes. The data showed that the activity of SOD was considerably lower in the OA group than the Ctrl group; however, it was significantly increased in the OA + UH group (Figure 5a). Compared with the Ctrl group, the contents of MDA were markedly increased in the OA group, but notably decreased by uridine administration (Figure 5b). The contents of GSH were decreased in the OA-treated cells, compared with the Ctrl cells; however, they were clearly increased in the OA + UM and OA + UH treatment groups when compared with the OA group (Figure 5c). Furthermore, the protein level of Nrf2 was decreased in the OA-treated group, but increased by uridine treatment (Figure 5d,e). The gene expression levels of *nqo1* and *ho1*, which were regulated by Nrf2 signaling pathway, were lower in the OA group relative to the Ctrl group, while the relative expression of *nrf2*, *nqo1,* and *ho1,* were increased in the uridine treatment group (Figure 5f). These data suggested that uridine could ameliorate the OA-induced oxidative stress by enhancing the antioxidative biomarkers and activating the Nrf2 signaling pathway.

In addition, different concentrations of uridine at 25 μM (H_2_O_2_ + UL), 125 μM (H_2_O_2_ + UM), and 250 μM (H_2_O_2_ + UH) were added to the 250 μM hydrogen peroxide (H_2_O_2_)-induced oxidative injury model in hepatocytes to define whether uridine could directly inhibit oxidative stress. We observed that the proliferation rate of cells was significantly decreased by H_2_O_2_ treatment, but clearly increased by uridine (Figure 5g). The activity of SOD was considerably lower in the H_2_O_2_ group than the Ctrl group; however, it was significantly increased in the H_2_O_2_ + UM and H_2_O_2_ + UH group (Figure 5h). Compared with the Ctrl group, the contents of MDA were markedly increased in the H_2_O_2_ group, but notably decreased by uridine administration (Figure 5i). The contents of GSH were decreased in the H_2_O_2_-treated cells; however, they were clearly increased in uridine-treated cells (Figure 5j). These results indicated that uridine could attenuate oxidative stress directly.

### 3.7. L. plantarum MR1 Supplementation Alters the Gut Microbiota Composition of Nile Tilapia

**Figure 6:**
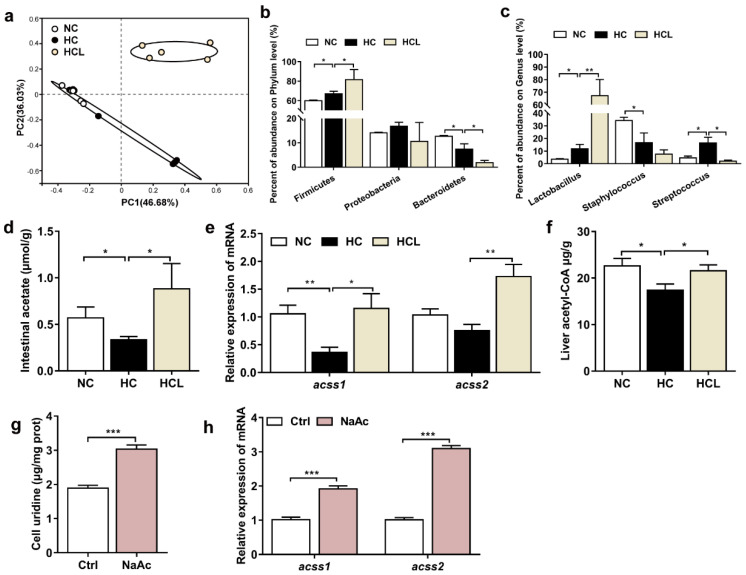
*L. plantarum* MR1 altered the gut microbiota composition. *L. plantarum* MR1-produced acetate promoted the uridine synthesis of Nile tilapia. (**a**) Principal coordinate analysis (PCoA) of the intestinal bacterial community. (**b**) Abundance of the gut bacteria at the phylum levels. (**c**) Abundance of the gut bacteria at the Genus levels. (**a**–**c**) *n* = 6 in NC and HC groups and *n* = 5 in HCL group. (**d**) Intestinal short-chain fatty acids (SCFAs) detection in Nile tilapia (*n* = 6). (**e**) Gene expression of *acss1* and *acss2* in the liver (*n* = 6). (**f**) Detection of the concentration of acetyl-CoA in the liver by HPLC (*n* = 6). (**g**) Analysis of uridine contents in the sodium acetate (NaAc)-treated hepatocytes (*n* = 6). (**h**) Gene expression of *acss1* and *acss2* in the hepatocytes (*n* = 6). Statistics were analyzed by one-way ANOVA with Tukey’s adjustment (**a**–**f**) or Student’s *t*-test (**g**,**h**), and presented as mean ± SEM (*, *p <* 0.05; **, *p <* 0.01; ***, *p <* 0.001).

To reveal the relationship between the addition of *L. plantarum* MR1 and the content of serum uridine, the intestinal bacterial composition and intestinal microbiota derived metabolites were detected. The composition of gut microbiota was characterized by 16S rRNA amplicon pyrosequencing. The principal coordinate analysis (PCoA) displayed that addition of *L. plantarum* MR1 changed the composition of gut microbiota (Figure 6a). The abundance of Firmicutes was increased in the HC group in contrast with the NC group and further increased in the HCL group (Figure 6b). The abundance of *Lactobacillus* belonging to Firmicutes was slightly increased in the HC group relative to the NC group, but markedly enriched by *L. plantarum* MR1 supplementation (Figure 6c). The bacteria metabolites were detected, and compared with the NC group. The intestinal acetate was significantly decreased in the HC group, but dramatically elevated in the HCL group (Figure 6d).

A previous study has reported that sodium acetate (NaAc) can be metabolized to acetyl-CoA, and then act as a substrate to promote the synthesis of uridine in endothelial cells [37]; however, whether intestinal microbiota-derived acetate could increase the uridine synthesis remains unknown. The gene expression levels of *acss1* and *acss2*, which were responsible for the synthesis of acetyl-CoA from acetate, were detected in the liver. Compared with the NC group, *ascc1* was significantly downregulated in the HC group, and the presence of *L. plantarum* MR1 increased the transcript levels of *ascc1* and *ascc2* (Figure 6e). The contents of acetyl-CoA in the liver were lower in the HC group than the NC group, but distinctly increased in the HCL group (Figure 6f). To detect whether acetate could directly promote the synthesis of uridine, 20 mM of NaAc was incubated with the primary hepatocytes of Nile tilapia, and the concentration of uridine was detected. The contents of uridine were significantly increased by the addition of NaAc (Figure 6g). The gene expression levels of *acss1* and *acss2* were both stimulated by the NaAc treatment in the hepatocytes (Figure 6h). Considered together, these data suggested that *L. plantarum* MR1 altered the composition of gut microbiota and increased the abundance of *Lactobacillus*. Moreover, gut microbiota-derived acetate could act as a substrate for the uridine synthesis in the liver of Nile tilapia.

## 4. Discussion

Host-microbiota mutualism is an integral part for the maintenance of health status. In addition, it is increasingly clear that the interaction between the host and intestinal microbiota has an impact on all tissues, in addition to the gut [38]. Beyond the fact that the components of bacteria have a direct influence on the host, intestinal microbiota can also metabolize dietary components to produce metabolites that may enter the circulation system to influence the host [39]. In the present study, we demonstrated that addition of *L. plantarum* MR1 altered the composition of gut microbiota and increased the contents of intestinal acetate to promote the production of uridine. We further detected higher contents of uridine in the NaAc-treated primary hepatocytes of the fish. The data suggested that the intestinal microbiota-derived acetate may promote the synthesis of uridine in the liver. Increased uridine may be responsible for the *L. plantarum* MR1 mediated beneficial effect, including protection against oxidative stress and lipid accumulation in the liver.

SCFAs are the most abundant dietary metabolites produced by gut microbiota. In addition, multiple studies indicated that they can regulate lipid and glucose metabolism in the host [40]. However, whether the microbiota and its metabolites can regulate the nucleotides synthesis of the host, as well as the mechanisms, are still unclear. It has been found that gut microbiome alteration is closely related to the change in the metabolism of nucleotides. For example, individual bat foraging on birds (by the great evening bat) had a higher abundance of Firmicutes and the Firmicutes–Bacteroidetes ratio, which may be associated with the metabolism of carbohydrates and nucleotides; however, the conclusion was drawn from the potential function prediction based on the sequencing data [41]. *L. plantarum* belongs to the phylum of Firmicutes, and we found a higher abundance of Firmicutes in the HCL group. It is well known that collapsing data to “phylum” level has generated a decade-long debate over controversial results of the relationship between the bacteria composition and host metabolic characteristics [42]. Therefore, elucidating the bacteria function will provide more solid evidence to understand the microbiota–host communications. We hypothesized that intestinal microbiota-derived acetate could be metabolized into acetyl-CoA to induce the synthesis of uridine in the liver. It has been reported that the loss of carnitine palmitoyl transferase 1 (CPT1) reduced acetyl-CoA, which originates from fatty acid and impaired de novo nucleotide synthesis, while supplementation with NaAc facilitated nucleotide synthesis in the endothelial cells [37]. Liver is generally considered as the major organ for the synthesis of uridine, which can enter the systemic circulation through the portal vein [43]. In the present study, we demonstrated that addition of sodium acetate in the primary hepatocytes of Nile tilapia could promote the uridine synthesis in vitro, and uridine may be responsible for exerting the antioxidant function and alleviating lipid accumulation directly. To our knowledge, this is the first study to show that intestinal bacteria-derived acetate contributed to the synthesis of uridine.

Uridine is the main pyrimidine nucleoside in the plasma of human and rodent. It is critical for RNA synthesis, glycogen synthesis, and many other cellular processes [44]. Moreover, it has been reported that uridine significantly decreases the accumulation of white adipose tissue and liver lipid by modifying gut microbiota composition in mice [45]. There are two possible mechanisms for the relationship between uridine and lipid metabolism. It has been found that uridine may alter the ratios of NAD+/NADH and NADP+/NADPH in the liver and modulate the protein acetylation profile to regulate lipid metabolism [46]. Furthermore, it has been found that inhibition of dihydroorotate dehydrogenase (DHODH) may cause micro-vesicular steatosis, which can be alleviated by the treatment with uridine; however, uridine has no effect on DHODH activity in vitro [46]. The exact regulatory mechanisms of uridine on glucose and lipid metabolism still remain unclear [44]. In the present study, the activation of AMPKα was increased in the HCL group, suggesting that uridine may reduce lipid deposition by activating the AMPKα signaling pathway [47].

More recently, uridine has been suggested to prevent the oxidative damage of lungs, which are caused by the coronavirus disease 2019 (COVID-19). The possible reason for this is the fact that uridine acts as the substrate of uridine-5′-diphosphate (UDP), which can prevent the excessive production of hydrogen peroxides [48]. Consistently, we proved that uridine could directly diminish H_2_O_2_-induced oxidative stress in the primary hepatocytes of Nile tilapia, suggesting that uridine had a resistance effect on oxidative stress. Furthermore, uridine could reduce lipid accumulation and oxidative stress of OA-induced damage in the hepatocytes.

## 5. Conclusions

In conclusion, these data demonstrate that addition of *L. plantarum* MR1 in high-carbohydrate diet alters the composition of intestinal microbiota, and thus increases the intestinal acetate which promotes the synthesis of uridine. Uridine exerts an antioxidant effect and decreases the hepatic lipid accumulation. Our study deepened the knowledge of gut microbiota and its metabolites, which are responsible for the alleviation of oxidative stress and lipid deposition. Overall, this suggests that gut microbiota-derived metabolites can act as a precursor substance for the metabolism of nucleotides to maintain the liver health in fish fed with high-carbohydrate diet.

## Figures and Tables

**Table 1:** Effect of *L. plantarum* MR1 on growth performance parameters, body composition indexes, and body composition contents of Nile tilapia.

Groups	NC	HC	HCL
Growth performance parameters			
IBW (g)	1.64 ± 0.05	1.67 ± 0.05	1.68 ± 0.05
FBW (g)	20.16 ± 0.60	22.01 ± 2.41 #	25.98 ± 2.22
WGR (%)	1089.00 ± 48.15	1250.77 ± 32.05 #	1391.08 ± 20.04 *
Body composition indexes (%)			
VSI (%)	12.31 ± 1.14	14.44 ± 1.45 ##	11.78 ± 0.78 ***
HSI (%)	2.85 ± 0.25	3.66 ± 0.58 #	2.93 ± 0.41 *
MFI (%)	0.59 ± 0.16	0.71 ± 0.39	0.52 ± 0.09
CI (%)	48.77 ± 1.61	47.12 ± 2.93	49.43 ± 1.73 *
Body composition contents (%)			
Moisture	72.31 ± 1.04	71.49 ± 1.21	72.42 ± 0.66
Total lipid	6.55 ± 0.75	8.23 ± 0.64 ##	6.92 ± 0.55 *
Total protein	14.67 ± 0.29	13.94 ± 0.58	14.16 ± 0.45
Ash	2.66 ± 0.47	3.09 ± 0.22	3.34 ± 0.27

IBW: Initial body weight (g); FBW: Final body weight (g); WGR: Weight gain rate (%); VSI: Visceral somatic index (%); HSI: Hepatosomatic index (%); MFI: Mesenteric fat index (%); CI: Carcass index (%). Statistics were analyzed by one-way ANOVA with Tukey’s adjustment and presented as mean ± standard error of the mean (SEM) (#, NC v.s. HC; *, HC v.s. HCL, #, *p* < 0.05; ##, *p* < 0.01; *, *p* < 0.05; ***, *p* < 0.001).

## Data Availability

The 16S rRNA gene sequence of L. plantarum MR1 was submitted to NCBI GenBank (https://www.ncbi.nlm.nih.gov/genbank/, accessed on 22 May 2022) with the accession number: OM535884. The microbiota raw data were deposited into the NCBI Sequence Read Archive (SRA) database (https://www.ncbi.nlm.nih.gov/sra, accessed on 22 May 2022) with accession number: PRJNA615286 and PRJNA805534. The metabolomic data were deposited and available at MetaboLights (https://www.ebi.ac.uk/metabolights/, accessed on 22 May 2022) under accession number: MTBLS4262. Data is contained within the article and supplementary materials.

## Supplementary Materials

The following supporting information can be downloaded at: https://www.mdpi.com/article/10.3390/antiox11071238/s1. Supplementary Methods, Table S1: Formulations of the diets; Table S2: Primers used for RT-qPCR expression analysis; Table S3: The antibodies information for immunofluorescence and Western blotting assay; Figure S1: *L. plantarum* MR1 inhibited lipid accumulation-induced liver inflammation in Nile tilapia; Figure S2: Uridine suppressed the OA-induced inflammation in the primary hepatocytes of Nile tilapia.

## Abbreviations

HC	high-carbohydrate diet
*L. plantarum*	lactobacillus plantarum
H&E	hematoxylin and eosin
ORO	oil red O
SCFAs	short-chain fatty acids
GPR	G-protein coupled receptor
FBW	final body weight
WGR	weight gain rate
VSI	visceral somatic index
HSI	hepatosomatic index
MFI	mesenteric fat index
CI	carcass index
AST	aspartate aminotransferase
ALT	alanine aminotransferase
PCoA	principal coordinate analysis
TG	triglyceride
NEFA	non-esterified free fatty acids
SOD	superoxide dismutase
MDA	malondialdehyde
GSH	reduced glutathione
H_2_O_2_	hydrogen peroxide
OA	oleic acid
NaAc	sodium acetate
