# Supplementary material for: Lactobacillus plantarum Ameliorates High-Carbohydrate Diet-Induced Hepatic Lipid Accumulation and Oxidative Stress by Upregulating Uridine Synthesis
Source: Antioxidants (Basel). 2022 Jun 24;11(7):1238. doi: 10.3390/antiox11071238 (PMC9312134; doi:10.3390/antiox11071238)
Supplement: Supplementary file 1 [file antioxidants-11-01238-s001.zip › antioxidants-1760292-supplementary.pdf]

Online Supplementary Materials

Lactobacillus plantarum ameliorates high-carbohydrate diet-induced hepatic lipid accumulation and oxidative stress by upregulating uridine synthesis

Rong Xu, et al.

Supplementary Methods

Bacteria identification and diet preparation.

One hundred and thirty-four bacterial isolates were purified from the gut of healthy Nile tilapia and cultured at 28°C in MRS broth medium (BS1137, Basebio). Twelve clusters were classified according to the amplified ribosomal DNA restriction analysis (ARDRA) as the previous study described [1]. A strain from the dominant cluster was randomly selected and designated as MR1. The genomic DNA of MR1 was extracted by using a bacterial DNA kit (DP302, Tiangen) according to the manufacturer's protocol. The 16S rRNA gene of MR1 was amplified using primers 27F (5'-AGAGTTTGATCCTGGCTCAG-3') and 1492R (5'-GGTTACCTTGTTACGACTT-3'). The PCR was performed by using the following program: 94°C for 5 min; 30 cycles at 94°C for 30 s, 55°C for 30 s and 72°C for 90 s; and 72°C for 10 min. The 16S rRNA gene was sequenced by Majorbio Bio-Pharm Technology Co. Ltd and blasted in GenBank (https://www.ncbi.nlm.nih.gov/genbank/). The 16S rRNA gene sequence of MR1 was affiliated to *Lactobacillus plantarum* and submitted to NCBI GenBank with the accession number: OM535884. *L. plantarum* MR1 was cultured at 28 °C in MRS agar medium (BS1138, Basebio) under anaerobic conditions by using the Anaeropack (Mitsubishi Gas Chemical Company, Inc., Japan) for 16 h. The bacteria quantity was determined by serial dilution and count on MRS agar plate. Stock of *L. plantarum* MR1 was mixed with glycerol, and stored at −80°C. The cells were harvested by centrifuging (12,000×g), washing twice with 1× phosphate buffer solution (PBS) and resuspending in PBS, and then mixed with the HC diet to reach a final concentration of 10^9 colony forming units (CFU)/g.

Sampling collection.

Before the sampling, fish were fasted for 12 h. Nine fish from each treatment were anesthetized by eugenol tranquilizers at 20 mg/L. Blood was collected from the caudal vein and centrifuged for serum preparation (3,500×g for 10 min). The serum was immediately frozen at −80°C for further analysis. Tissue samples were collected to perform biochemical and molecular biological assays. The intestinal contents were collected for measurement of SCFAs. The weight gain rate (WGR), visceral index (VSI), hepatosomatic index (HSI), mesenteric fat index (MFI) and carcass index (CI) were calculated using the following formulae:

WGR (%) = 100 × (Final fish weight-initial fish weight) / initial fish weight

VSI (%) = 100 × (Visceral weight / body weight)

HSI (%) = 100 × (Liver weight / body weight)

MFI (%) = 100 × (Mesenteric fat weight / body weight)

CI (%) = 100 × (Carcass weight / body weight)

16S rRNA amplicon sequencing.

Intestinal bacterial genomic DNA was purified using an E.Z.N.A.® Soil DNA Kit (D5625, Omega,) according to the manufacturer's instructions. The extracted DNA was checked on 1% agarose gel, and DNA concentration and purity were determined with a Nano Drop 2000 UV-vis spectrophotometer (Thermo Fisher Scientific, USA). The V3-V4 region of bacterial 16S rRNA gene was amplified with primers 338F (5'-ACTCCTACGGGAGGCAGCAG-3') and 806R (5'-GGACTACHVGGGTWTCTAAT-3'). The PCR amplification of 16S rRNA gene was performed as follows: initial denaturation at 95°C for 3 min, followed by 27 cycles of denaturing at 95°C for 30 s, annealing at 55°C for 30 s and extension at 72°C for 45 s, and single extension at 72°C for 10 min, and end at 4°C. 20 μL PCR mixtures contain 5 × *TransStart* FastPfu buffer 4 μL, 2.5 mM dNTPs 2 μL, forward primer (5 μM) 0.8

μL, reverse primer (5 μM) 0.8 μL, *TransStart* FastPfu DNA Polymerase 0.4 μL and template DNA 10 ng. PCR reactions were performed in triplicate. The purified amplicons were pooled in equimolar and sequenced on an Illumina MiSeq PE300 platform/NovaSeq PE250 platform (Illumina, USA) by Majorbio Bio-Pharm Technology Co. Ltd. (Shanghai, China). The raw reads were deposited into the NCBI. The microbiota raw data were deposited into the NCBI Sequence Read Archive (SRA) database (https://www.ncbi.nlm.nih.gov/sra) with accession number: PRJNA615286 and PRJNA805534.

Operational taxonomic units (OTUs) with 97% similarity cutoff were clustered using UPARSE v.7.1, and chimeric sequences were identified and removed. The taxonomy of each OTU representative sequence was analyzed by RDP Classifier v.2.2 against the 16S rRNA database using confidence threshold of 0.7. Thirty-four OTUs were selected for heat-map analysis based on: (i) the abundances of these OTUs were higher than 0.01% in each sample and (ii) the abundance of these OTUs was significantly different among groups by using Turkey's adjustment analysis. Taxonomic richness and diversity estimators were determined using the Mothur software. Principal coordinates analysis and heat-map analysis were performed in a MATLAB R2016a environment.

Measurement of SCFAs.

The bacteria solution or intestinal contents was extracted with 50% sulphuric acid and vortexed for 10 min. The extracted samples were centrifuged at 4°C (12,000×g for 10 min) and filtered with membranes (0.22 μm pore size) before analysis by a gas chromatography (GC)-2030 with an autosampler (Shimadzu, Japan) and a Stabilwax-DA capillary column (30 m, 0.25 mm i.d., 0.25 μm film thickness; Shimadzu). For the chromatographic separation, nitrogen was used as carrier gas at a constant flow rate of 1 mL/min; hydrogen was utilized as auxiliary gas with the flame ionization detection (FID) temperature at 200°C, the injection port temperature at 220°C. For the analysis,

1 μL of sample was detected by the following temperature gradient: temperature was set to 80°C initially, then increased to 180°C at 5°C per min and maintained for 2 min.

Mass Spectrometry.

Serum samples of 100 μL were extracted using a 400 μL methanol: water (4:1, *v/v*) solution with 0.02 mg/mL L-2-chlorophenylalanin as internal standard. The mixture was settled at −10°C and treated by high throughput tissue crusher at 50 Hz for 6 min, then ultrasound at 40 kHz for 30 min at 5°C. The samples were placed at −20°C for 30 min to precipitate proteins. After centrifugation at 4°C (13,000×g for 15 min), the supernatants were transferred to sample vials for liquid chromatography-mass spectrometry (LC-MS) analysis. Quality control sample (QC) was prepared by mixing equal volumes of all samples. The QC samples were used to monitor the deviation in the analysis results of pooled sample mixtures and to compare this deviation with the error caused by the analyzer.

Chromatographic separation of the metabolites was performed on a Thermo UHPLC system equipped with an ACQUITY UPLC HSS T3 (100 mm × 2.1 mm i.d., 1.8 μm, USA) column. The mobile phases consisted of 0.1% formic acid in water: acetonitrile (95:5, *v*/*v*) (mobile phase A) and 0.1% formic acid in acetonitrile: isopropanol: water (47.5: 47.5: 5, *v*/*v*/*v*) (mobile phase B). The mobile phase gradient changed according to the following conditions: 0-3.5 min, 24.5% B; 3.5-5 min, 65% B; 5-7.4 min, 100% B; 7.4-7.6 min, 51.5% B; 7.6-10 min, 0% B for equilibrating the systems. The sample injection volume was 2 μL, and the flow rate was 0.4 mL/min. The column temperature was maintained at 40°C. During the period of analysis, all these samples were stored at 4°C. The mass spectrometric data was collected using a Thermo UHPLC-Q Exactive HF-X Mass Spectrometer equipped with an electrospray ionization (ESI) source operating in either positive or negative ion mode. The ion-spray voltage floating was set to 3.5 kV. The sampling normalized collision energy ranged from 20 V to 60 V rolling for MS/MS. Data acquisition was performed with the Data Dependent

Acquisition (DDA) mode. The detection was carried out over a mass range of 70-1050 m/z. The metabolomic data were deposited and available at MetaboLights (https://www.ebi.ac.uk/metabolights/) under accession number: MTBLS4262.

After LC-MS analyses, the raw data were imported into the Progenesis QI v.2.3 (Nonlinear Dynamics, USA) for peak detection and alignment. A multivariate statistical analysis was performed using ropls v.1.6.2 (http://bioconductor.org/packages/release/bioc/html/ropls.html) R package from Bioconductor. Statistical significance among groups were selected with VIP (variable importance for the projection) value more than 1 and p value less than 0.05. Differential metabolites among groups were summarized, and mapped into their biochemical pathways by metabolic enrichment and pathway analysis based on database search (KEGG, https://www.genome.jp/kegg/).

Cell experiment.

To confirm the effects of uridine, 250 μM oleic acid (OA) (HY-N1446, MCE) was added to the hepatocytes and then supplemented with uridine (V900421, Sigma-Aldrich) at concentration of 25 μM (OA+UL), 125 μM (OA+UM) and 250 μM (OA+UH) for 24 h. The cell proliferation rate was tested by CCK-8 kit (GK10001, Glpbio). Lipid droplets in the hepatocytes were fixed with 4% paraformaldehyde followed by staining with 10 μg/mL BODIPY 493/503 (D3922, Invitrogen) for 15 min at room temperature. The images were captured by using the Nikon fluorescence microscopy (Nikon).

To verify the antioxidant effect of uridine, 250 μM hydrogen peroxide (H_2O_2)-induced oxidative damage model was established and supplemented with uridine at concentration of 25 μM (H_2O_2+UL), 125 μM (H_2O_2+UM) and 250 μM (H_2O_2+UH) for 24 h.

Uridine and acetyl-CoA detection by HPLC.

Uridine and acetyl-CoA were detected by high performance liquid chromatography (HPLC) (LC-20AT, Shimadzu, Japan) with a tandem double plunger. The separation was achieved on the Shim-pack GIST 5μm C18 column (4.6×250 μm, 5μm particle size, Shimadazu®). The column temperature was maintained at 40°C, and the injection volume was set at 10 μL.

For analysis of serum uridine, the samples were extracted with 1 mL of 9:1 ethyl acetate: isopropyl alcohol (*v*: *v*) by vortex-mixing for 5 min. Each sample was centrifuged at 4°C (12,000×g for 10 min). The supernatants were dried by nitrogen gas. The residue was dissolved in 50 μL 0.2% acetic acid, vortexed for 5 min and filtered with 0.22 μm pore size membrane. The supernatant of each sample and the uridine standard solutions were injected into the HPLC system. The mobile phase (A) contained 0.2% acetic acid in water. The mobile phase (B) contained 0.2% acetic acid in acetonitrile. The flow rate was 1 mL/min in equal gradient mode: (A): (B) (*v*: *v*) =85%: 15%.

For analysis of liver acetyl-CoA, 40 mg liver sample was homogenized with 5% perchloric acid containing 50 μM dithiothreitol as 1:10 (*w*/*v*), vortexed for 5 min and placed on ice for 20 min. Each sample was centrifuged at 4°C (12,000×g for 10 min), and the supernatant was filtered before analysis. The supernatant of each sample and acetyl-CoA (A2181, Sigma-Aldrich) standard solutions were injected into the HPLC system. The mobile phase (A) was 60% methyl alcohol. The mobile phase (B) contained 100 mM sodium dihydrogen phosphate and 75 mM NaAc in 6: 94 acetonitrile: water (*v*: *v*), and the pH was adjusted to 4.6. The flow rate was 1 mL/min in equal gradient mode: (A): (B) (*v*: *v*) =10%: 90%.

Supplementary Tables

Supplementary Table S1. Formulations of the diets.

Ingredients	NC	HC	HCL
Casein (g/kg)	320	320	320
Gelatin (g/kg)	80	80	80
Soybean oil (g/kg)	70	70	70
Corn starch (g/kg)	300	450	450
Vitamin premix [1] (g/kg)	10	10	10
Mineral premix [2] (g/kg)	10	10	10
$Ca(H_2PO_4)_2$ (g/kg)	10	10	10
Carboxymethylcellulose (g/kg)	25	25	25
Cellulose (g/kg)	167.75	17.75	17.75
Choline chloride (g/kg)	5	5	5
Dimethyl-β-propiothetin (g/kg)	2	2	2
Butylated hydroxytoluene (g/kg)	0.25	0.25	0.25
L. plantarum MR1 (CFU/g)	0	0	10^9

1. Mixed vitamin (mg or IU/kg): 500,000 I.U. Vitamin A, 50,000 I.U. Vitamin D3, 2500 mg Vitamin E, 1000 mg Vitamin K3, 5000 mg Vitamin B1, 5000 mg Vitamin B2, 5000 mg Vitamin B6, 5000 mg Vitamin B12, 25,000 mg Inositol, 10,000 mg Pantothenic acid, 100,000 mg Cholin, 25,000 mg Niacin, 1000 mg Folic acid, 250 mg Biotin, 10,000 mg Vitamin C.

2. Mixed minerals (g/kg): 469.3 g KH_2PO_4; 314.0g NaCl; 147.4 g $MgSO_4•7H_2O$; 49.8 g NaCl; 10.9 g Fe (II) gluconate; 3.12 g $MnSO_4•H_2O$; $ZnSO_4•7H_2O$; 0.62 g $CuSO_4•5H_2O$; 0.16 g KI; 0.08 g $CoCl_2•6H_2O$; 0.06 g NH_4 molybdate; 0.02 g $NaSeO_3$.

Supplementary Table S2. Primers used for RT-qPCR expression analysis.

Gene	Primer sequence (5’-3’)	Accession number
accα	F: TAGCTGAAGAGGAGGGTGCAAGA	XM_025910668.1
	R: AACCTCTGGATTGGCTTGAACA	
acss1	F: AGGTGACCGAGTTGCGATTT	XM_005462220.4
	R: TACCTGCCTGGGTTCGGATA	
acss2	F: GCATCATGCTGAAGCACCTG	XM_005477775.4
	R: TTTTCCTGCTGCAGATCGGG	
atgl	F: GACACATGCTGCAAAGCACT	XM_003440346.5
	R: ACCAGGACGTTTTCTCCGTC	
cpt1a	F: TTTCCAGGCCTCCTTACCCA	XM_013268638.3
	R: TTGTACTGCTCATTGTCCAGCAGA	
dgat2	F: GCTTGAATTCTGTCACCCTGAAGA	XM_003458972.5
	R: ACCTGCTTGTAGGCGTCGTTCT	
ef1-α	F: ATCAAGAAGATCGGCTACAACCCT	AB075952.1
	R: ATCCCTTGAACCAGCTCATCTTGT	
fas	F: TCATCCAGCAGTTCACTGGCATT	XM_003454056.5
	R: TGATTAGGTCCACGGCCACA	
fatp1	F: ATGCCAAAGGCAGCTGTAGT	XM_003450007.5
	R: CGTCGAATCTTCTGAGCGGA	
ho1	F: AAGAGAGTGATCGTCCCTGC	XM_013270165.3
	R: GATCACCGAGGTAGCGAGTG	
hsl	F: GCGCTCTGAGTGTCTTGCTA	XM_003447253.4
	R: ACTATGTGTACAGGCGGCAG	
il-1β	F: GGAAATTGCCACCGCAAAGT	XM_005457887.3
	R: GACAGGACGACAATCAGACCA	
nf-κb	F: GCCTCTGAGCAGGCTGTAAA	XM_005467394.3
	R: GTGTAGCTGACACACGACCA	

nqo1	F: ACCTGCGTTCCAACTACCTG	XM_003455884.4
	R: GCTTCATAGCGAGGGTTCGT	
nrf2	F: CCTGGAGGTCTTTGGCATGT	XM_003447296.5
	R: ATCCGTTGACTGCTGAAGGG	
pparα	F: GTTCCTCAAGAGTCTCCGCC	XM_019346353.2
	R: AAAGAGCTAGGTCGCTGTCG	
pparγ	F: TGGACTACACAAACATGCACAGC	XM_019358463.2
	R: CACGGGACTATCTGAGTACTGTGGA	
tnfα	F: CCAACGCTGGTCTCACTCAT	XM_013266975.3
	R: GTTCTCAGTCTGTCCCCAGC	
β-actin	F: AGCCTTCCTTCCTTGGTATGGAAT	XM_003443127.5
	R: TGTTGGCGTACAGGTCCTTACG	

accα: acetyl-CoA carboxylase alpha; *acss1*: acetyl-coenzyme synthase 1; *acss2*: acetyl-coenzyme synthase 2; *atgl*: adipose triglyceride lipase; *cpt1a*: carnitine palmitoyl transferase 1a; *dgat2*: diacylglycerol acyltransferase 2; *ef1α*: elongation factor 1 alpha; *fas*: fatty acid synthase; *fatp1*: fatty acid transport protein 1; *ho1*: heme oxygenase 1; *hsl*: hormone-sensitive lipase; *il-1β*: interleukin-1 beta; *nf-κb*: nuclear factor-kappa b; *nqo1*: NAD(P)H: quinone oxidoreductase 1; *nrf2*: nuclear factor erythroid 2-related factor 2; *pparα*: peroxisome proliferator-activated receptor alpha; *pparγ*: peroxisome proliferator-activated receptor gamma; *tnfα*: tumor necrosis factor alpha;*β-actin*: beta-actin.

Supplementary Table S3. The antibodies information for immunofluorescence and western blotting assay.

Antibodies name	Source	Identifier
cleaved IL-1β (Asp116)	Affinity	Cat # AF4006
GAPDH	Abways	AB0036
NF-κB p65	Cell Signaling Technology	Cat # AF5006
Nrf2	Abways	Cat # CY5136
p-AMPKα (Thr172)	Cell Signaling Technology	Cat # 2531
p-NF-κB p65 (Ser536)	Affinity	Cat # AF2006
anti-Rabbit IgG	Li-Cor Biosciences Corporate	Cat # 926-32211

Supplementary Figures

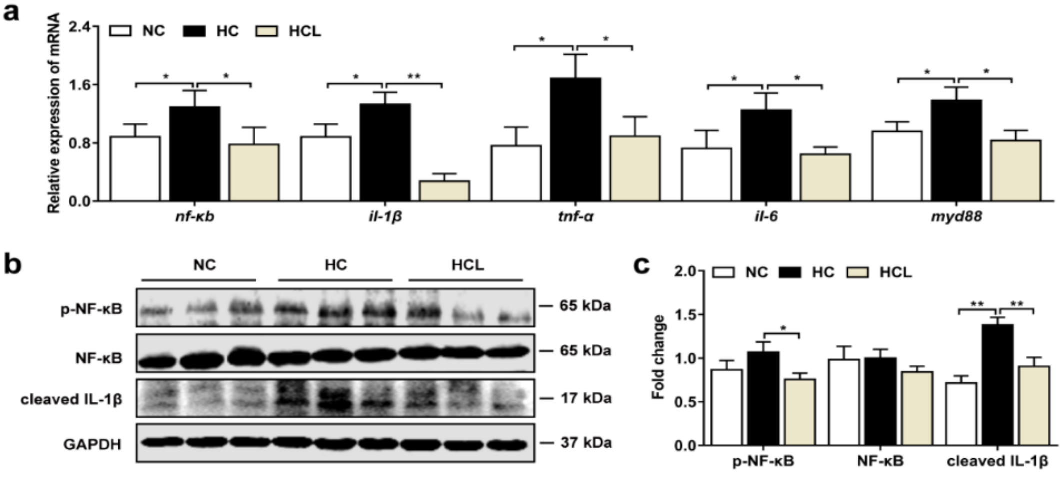

Figure S1 *L. plantarum* MR1 inhibited lipid accumulation-induced liver inflammation in Nile tilapia. (a) Expression of mRNA markers for inflammatory response (n=6). (b) The protein expression of phosphorylated-nuclear factor-kappa B (p-NF-κB), NF-κB and cleaved interleukin-1 beta (IL-1β) in liver tissues (n=3). (c) The protein of p-NF-κB, NF-κB and cleaved IL-1β was quantified and normalized by GAPDH (n=3). Statistics were analyzed by one-way ANOVA with Turkey's adjustment and presented as mean ± SEM (*, $P < .05$; **, $P < .01$).

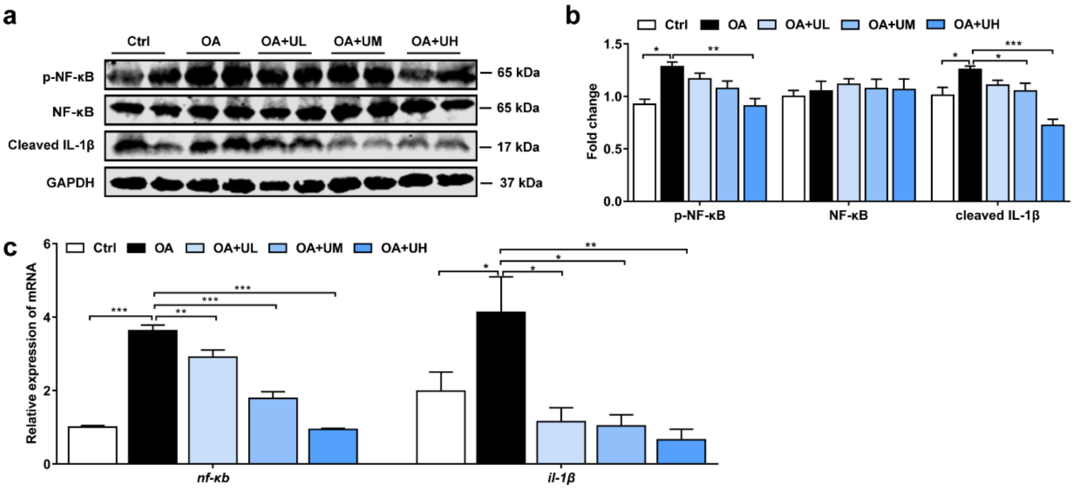

Figure S2 Uridine suppressed the OA-induced inflammation in the primary hepatocytes of Nile tilapia. (a) The protein expression of p-NF-κB, NF-κB and cleaved IL-1β in the primary hepatocytes (n=4). (b) Quantitation of p-NF-κB, NF-κB and

cleaved IL-1β levels normalized to GAPDH levels from blots shown in (a) (n=4). (c) Relative expression of mRNA involves in inflammatory signaling pathway in the hepatocytes (n=6). Statistics were analyzed by one-way ANOVA with Turkey's adjustment and presented as mean ± SEM (*, $P < .05$; **, $P < .01$, ***$P < .001$).
